# Dual Activation of the Bile Acid Nuclear Receptor FXR and G-Protein-Coupled Receptor TGR5 Protects Mice against Atherosclerosis

**DOI:** 10.1371/journal.pone.0108270

**Published:** 2014-09-19

**Authors:** Shinobu Miyazaki-Anzai, Masashi Masuda, Moshe Levi, Audrey L. Keenan, Makoto Miyazaki

**Affiliations:** Division of Renal Diseases and Hypertension, University of Colorado Denver, Aurora, Colorado, United States of America; Beckman Research Institute of City of Hope, United States of America

## Abstract

Bile acid signaling is a critical regulator of glucose and energy metabolism, mainly through the nuclear receptor FXR and the G protein-coupled receptor TGR. The purpose of the present study was to investigate whether dual activation of FXR and TGR5 plays a significant role in the prevention of atherosclerosis progression. To evaluate the effects of bile acid signaling in atherogenesis, ApoE^−/−^ mice and LDLR^−/−^ mice were treated with an FXR/TGR5 dual agonist (INT-767). INT-767 treatment drastically reduced serum cholesterol levels. INT-767 treatment significantly reduced atherosclerotic plaque formation in both ApoE^−/−^ and LDLR^−/−^ mice. INT-767 decreased the expression of pro-inflammatory cytokines and chemokines in the aortas of ApoE^−/−^ mice through the inactivation of NF-κB. In addition, J774 macrophages treated with INT-767 had significantly lower levels of active NF-κB, resulting in cytokine production in response to LPS through a PKA dependent mechanism. This study demonstrates that concurrent activation of FXR and TGR5 attenuates atherosclerosis by reducing both circulating lipids and inflammation.

## Introduction

In addition to their role in the formation of intestinal micelles, bile acids serve as signaling molecules through two major receptors, Farnesoid X Receptor (FXR) and TGR5. FXR is a nuclear receptor that is activated by bile acids such as chenodeoxycholic acid [1]–[3]. FXR is predominantly expressed in the liver, kidneys and intestine, and controls lipid and carbohydrate homeostasis [4]–[5]. Recent studies show that FXR activation by select agonists inhibits atherosclerosis development [6]–[7]. TGR5 (also designated as GPBAR1 or M-BAR) is a G-protein coupled bile acid receptor highly expressed in the intestine and gallbladder [8]–[9]. TGR5 is activated by both primary and secondary bile acids, but demonstrates the highest affinity for lithocholic acid (LCA) [8]. TGR5 mediates several biological effects of bile acids including a hypermetabolic effect, stimulation of gallbladder filling, and improved insulin sensitivity [10]–[12]. TGR5 is abundantly expressed in CD14-positive monocytes and macrophages, where its activation mediates immunosuppressive effects [8]. TGR5 activation by bile acids in monocytes, alveolar macrophages and Kupffer cells attenuates phagocytosis and cytokine production in response to lipopolysaccharides (LPS) in a cAMP-dependent manner [8],[13]–[15]. A recent study showed that pharmacological activation of TGR5 elicits anti-atherogenic effects by reducing macrophage inflammation and lipid uptake [13].

Several bile acids and their analogues have been reported to elicit anti-atherogenic effects through different mechanisms [7], [16]–[17]. We hypothesized that dual activation of FXR and TGR5 is effective in the prevention of atherosclerotic formation. In this study, we examined the pharmacologic effects of simultaneous activation of TGR5 and FXR on atherosclerotic plaque formation using a novel FXR and TGR5 dual agonist, 6α-ethyl-24-nor-5β-cholane-3α,7α, 23-triol-23 sulfate sodium salt (INT-767). Our present study demonstrates that dual activation of FXR and TGR5 strongly alleviates atherosclerotic formation primarily by reducing circulating lipids and reducing inflammation though the inactivation of NF-κB via a protein kinase A-dependent manner.

## Methods

### Animals

ApoE^−/−^ and LDLR^−/−^ mice on the C57BL/6J background were obtained from the Jackson Laboratory. Eight-week-old ApoE^−/−^ and LDLR^−/−^ mice were fed a Western diet (TD88137) containing INT-767 (30 mg/kg body weight) [18] for 12 weeks and 16 weeks, respectively. Eight animals per group were used for all experiments. Males were used because they are more susceptible to atherosclerosis than females. All animals were euthanized by isoflurane overdose after a 4 hour fasting period. Animal experiments were approved by the Institutional Animal Care and Research Advisory Committee of the University of Colorado at Denver. INT-767 was kindly provided by Intercept Pharmaceuticals Inc. (New York, NY).

### Histological and biochemical analysis

En face and histological analyses in the aortic sinus were performed as we previously described [19]–[20]. Immunofluorescence analysis for CD68 and MCP1 in the aortic root was performed using a Life Technologies EVOS *fluorescence* microscope as we described previously [21]. FPLC analysis was performed as previously described [22]. Fasted serum lipids and fast performance liquid chromatography samples were quantified using commercially available kits [22]. Serum bile acid levels were determined using an Applied Biosystems 3200 qTRAP LC-MS/MS according to a method previously described [23]. Serum inflammatory cytokine levels were measured using a commercially available ELISA kit (Meso Scale Discovery).

### Electrophoretic mobility shift assay

Electrophoretic mobility shift assay (EMSA) analysis was performed as previously described [24]. The DNA binding activity of NF-κB was assayed according to the protocol from Promega Corp. Briefly, the oligo with NF-κB consensus binding element (Promega) was end-labeled by T4 polynucleotide kinase (Promega) using [γP32]-ATP (BioRad). Thirty µg of total tissue extract was isolated from the aorta or nuclear extract from macrophages using 1X passive lysis buffer (Promega) was mixed with radio-labeled oligo for binding. Unlabeled cold probe was used to compete with the radio-labeled probe to show binding specificity. The reaction mixture was loaded to 5% polyacrylamide gel under non-denaturing conditions and separated by electrophoresis at 4°C. The gel was then dried and exposed to X-ray film to visualize the binding of NF-κB onto the radio-labeled probe. The binding specificity was shown by blockade of binding with excessive competitive cold probe, and the position of NF-κB p65/50 complex was confirmed using anti-p65 and -p50 antibodies from Cell Signaling Technology (Figure S1).

### Cell culture

J774.2 and Raw294.7 macrophages were pre-treated with 10 µg/ml INT-767 for 1 hour in the presence of a PKA inhibitor (Rp-8-Br-cAMPS, Santa Cruz Biotechnology), and then incubated with 100 ng/ml LPS. For FXR and TGR5 overexpression, Raw294.7 macrophages were transfected with pcDNA3 containing either FXR or TGR5. The stable cells expressing either FXR or TGR5 were selected with G418 (250 µg/ml).

### Statistical analysis

Data were collected from more than two independent experiments and reported as the means ± S.E.M. Statistical analysis for two-group comparison was performed using the Student's *t* test, or one-way ANOVA with a Newman-Keuls post-hoc test for multi-group comparison. Significance was accepted at *P*<0.05.

## Results

### INT-767 reduces serum cholesterol and triglyceride levels in ApoE^−/−^ and LDLR^−/−^ mice

**Figure 1:**
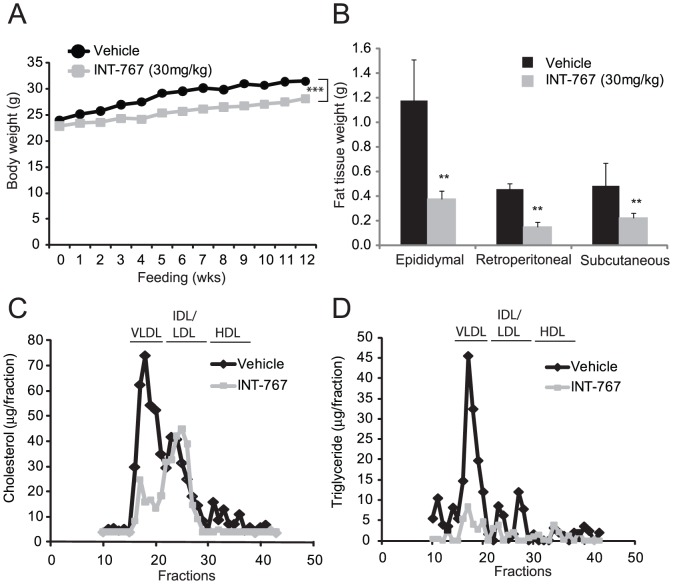
INT-767 reduces high fat-induced adiposity and hyperlipidemia in ApoE^−/−^ mice. Mice were treated with INT-767 as indicated in the *Methods* section. A) Body weight of ApoE^−/−^ mice treated with INT-767. B) White fat weight of ApoE^−/−^ mice. C) Cholesterol distribution in the lipoproteins of ApoE^−/−^ mice treated with INT-767. D) Triglyceride distribution in the lipoproteins of mice treated with INT-767. E) Body weight of LDLR^−/−^ mice treated with INT-767. F) Cholesterol distribution in the lipoproteins of LDLR^−/−^ mice treated with INT-767.

**Figure 2:**
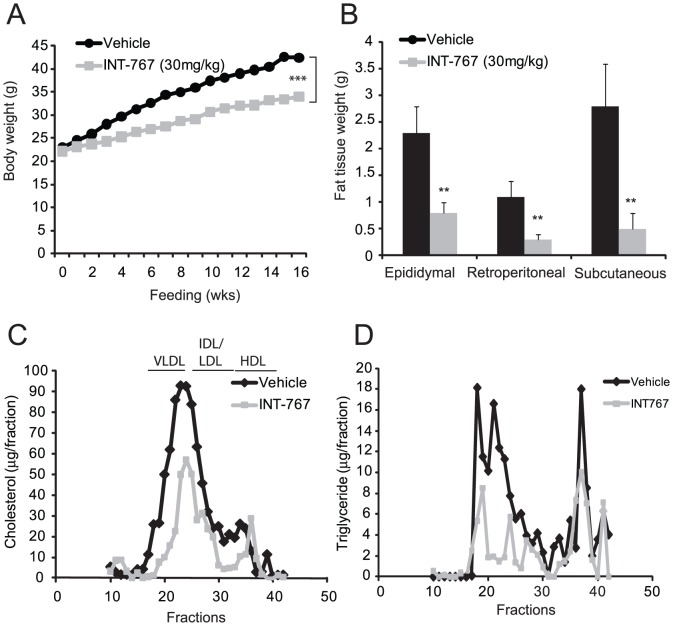
INT-767 reduces high fat-induced adiposity and hyperlipidemia in LDLR^−/−^ mice. Mice were treated with INT-767 as indicated in the *Methods* section. A) Body weight of LDLR^−/−^ mice treated with INT-767. B) White fat weight of ApoE^−/−^ mice. C) Cholesterol distribution in the lipoproteins of LDLR^−/−^ mice treated with INT-767. D) Triglyceride distribution in the lipoproteins of mice treated with INT-767. **P<0.001 and ***P<0.001.

To study the role of bile acid signaling in the regulation of atherogenesis, we first treated two commonly used mouse models of atherosclerosis, ApoE^−/−^ and LDLR^−/−^ mice, with INT-767. Eight-week-old ApoE^−/−^ mice were fed a Western diet alone (TD88137, Harlan Teklad) or a Western diet supplemented with INT-767 (30 mg/kg/day) for 12 weeks. ApoE^−/−^ mice treated with INT-767 showed a significant decrease in body weight (Table 1 and Fig. 1A). Consistently, the weights of major white adipose tissues (epididymal, retroperitoneal and subcutaneous) were all significantly reduced in mice treated with INT-767 (Fig. 1B and Table 1). In ApoE^−/−^ mice, INT-767 treatment significantly reduced serum total cholesterol and triglyceride levels compared with the control group (Table 1). Lipoprotein profile analysis showed reduced cholesterol levels in the VLDL and HDL fractions but not LDL and IDL fractions and reduced triglyceride levels in the VLDL fractions in ApoE^−/−^ mice treated with INT-767 (Fig. 1C and 1D). Serum glucose was reduced by INT-767 treatment (Table 1). The lipid-lowering effect of FXR and TGR5 dual activation was also examined in LDLR^−/−^ mice. Similar to ApoE^−/−^ mice, INT-767 attenuated the weight gain of LDLR^−/−^ mice induced by feeding a Western diet (Fig. 2B and Table 1). INT-767 reduced high fat feeding-induced adiposity in LDLR^−/−^ mice (Fig. 2B). Serum total cholesterol, triglyceride, and glucose levels were significantly reduced by INT-767 treatment (Table 2). INT-767 significantly reduced serum glucose in LDLR^−/−^ mice. Lipoprotein profile analysis showed reduced VLDL and LDL/IDL-cholesterol in mice treated with INT-767 (Fig. 2C). INT-767 treatment drastically reduced VLDL-triglyceride levels in LDLR^−/−^ mice.

**Table 1:** Serum lipid levels in ApoE^−/−^ mice treated with INT-767 for 12 weeks.

Treatment			vehicle			INT-767	
Dose	unit		-			30 mg/kg	
Total cholesterol	mg/dl	1561.4	±	16.5	**587.8** *	±	33.8
Triglyceride	mg/dl	172.1	±	13.4	**96.6** *	±	3.1
Glucose	mg/dl	190.5	±	22.6	**148.2** *	±	10.3
Body weight	g	32.3	±	0.8	**26.9** *	±	0.4
Food intake	g/day	2.5	±	0.4	2.4	±	0.3

Eight-week-old mice were fed a Western diet containing INT-767 for 12 weeks.

Blood was drawn after 4 hours of fasting.

Data expressed as Mean ± SEM.

*p<0.05 vs. ApoE^−/−^ mice with vehicle.

**Table 2:** Serum lipid levels in LDLR^−/−^ mice treated with INT-767.

Treatment			vehicle			INT-767	
Total cholesterol	mg/dl	1595.0	±	23.2	358.2*	±	4.8
Triglyceride	mg/dl	288.5	±	6.5	100.4*	±	2.0
Glucose	mg/dl	208.6	±	2.2	160.0*	±	3.2
Body weight	g	38.1	±	0.6	32.4*	±	0.4
Food intake	g/day	2.5	±	0.1	2.6	±	0.2

Eight-week-old mice were fed a Western diet containing INT-767 for 16 weeks.

Blood was drawn after 4 hours of fasting.

Data expressed as Mean ± SEM. *p<0.05 vs. LDLR^−/−^ mice with vehicle.

### INT-767 abolishes hepatic CYPB1 expression, leading to lack of serum cholic acid and deoxycholic acid, but not chenodeoxycholic acid

**Figure 3:**
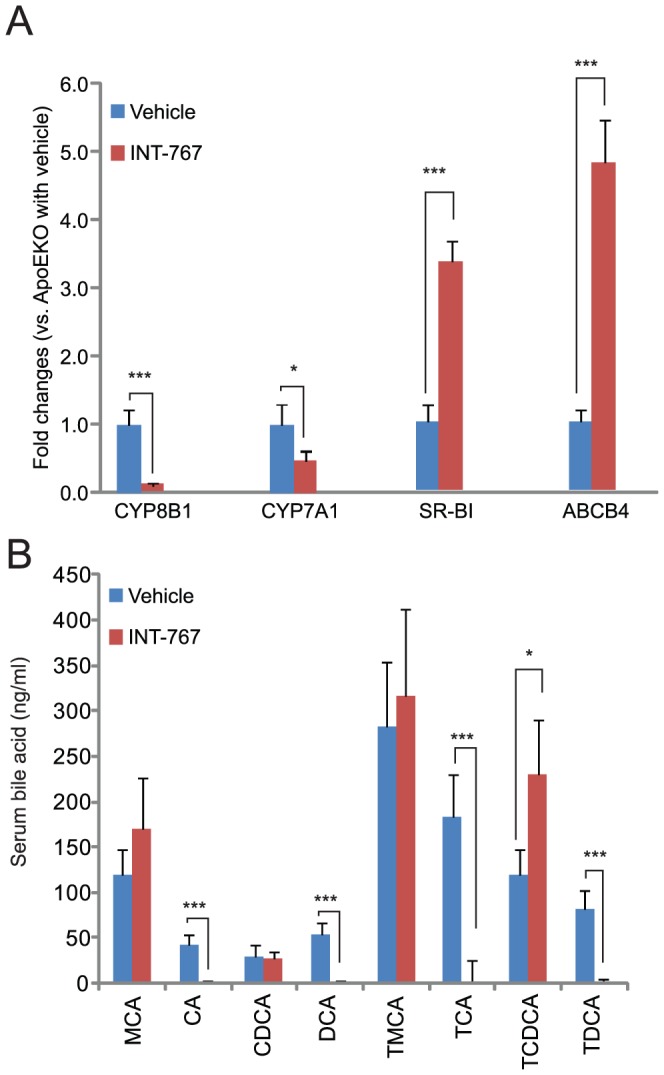
INT-767 reduces cholic acid and its metabolites but not chenodeoxycholic acid though the reduction of hepatic CYP7A1 and CYP8B1 expression in ApoE^−/−^ mice. A) mRNA levels of hepatic FXR targets in ApoE^−/−^ mice treated with INT-767. B) Serum bile acid content in ApoE^−/−^ mice treated with INT-767. CA, cholic acid; MCA, muricholic acid; DCA, deoxycholic acid; CDCA, chenodeoxycholic acid; T, tauro. *P<0.05 and ***P<0.001.

To determine whether INT-767 treatment activates hepatic FXR, we analyzed mRNA levels of FXR targets such as CYP7A1, CYP8B1, SR-BI and ABCB4 in the livers of ApoE^−/−^ mice. INT-767 treatment strongly reduced levels of hepatic CYP8B1 and CYP7A1 mRNA whereas SR-BI and ABCB4 levels were increased (Fig. 3A). Since INT-767 strongly inhibited the expression of CYP8B1 and CYP7A1, which are enzymes involved in bile acid synthesis, we analyzed levels of circulating bile acids using a liquid-chromatography mass spectrometry (LC-MS/MS). Interestingly, INT-767 drastically reduced serum cholic acid, deoxycholic acid and its tauro-conjugated metabolites. In contrast, chenodeoxycholic acid and its tauro-conjugated metabolites were unchanged or increased in the serum of ApoE^−/−^ mice (Fig. 3B) and LDLR^−/−^ mice (data not shown).

### INT-767 attenuates atherosclerotic plaque formation in ApoE^−/−^ and LDLR^−/−^ mice

**Figure 4:**
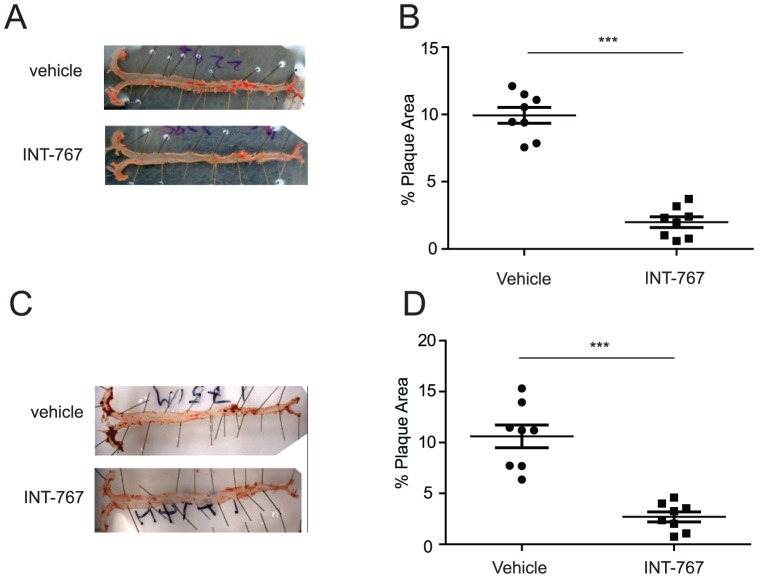
INT-767 inhibits the development of aortic lesions in ApoE^−/−^ and LDLR^−/−^ mice. Atherosclerotic lesions were quantified by en face analysis. A) Representative picture of en face analysis of atherosclerosis in ApoE^−/−^ mice treated with INT-767. B) Quantification of en face analysis in ApoE^−/−^ mice treated with INT-767 for 12 weeks. C) Representative picture of en face analysis of atherosclerosis in LDLR^−/−^ mice treated with INT-767. D) Quantification of en face analysis in LDLR^−/−^ mice treated with INT-767 for 16 weeks. ***P<0.001.

Atherosclerotic lesions in ApoE^−/−^ mice were quantified by en face analysis of aortas after 12 weeks of feeding a Western diet in the presence or absence of INT-767. Quantification of Sudan IV-stained en face preparations of aortas revealed a significant reduction in atherosclerosis in mice treated with INT-767. INT-767 treatment reduced atherosclerotic plaques by 81% (Fig. 4A and 4B) compared with vehicle treatment. Similarly, LDLR^−/−^ mice receiving an INT-767 agonist for 16 weeks showed a 72% reduction in atherosclerotic lesions (Fig. 4C and 4D) [13].

### INT-767 reduced macrophage infiltration and inflammation in ApoE^−/−^ mice

**Figure 5:**
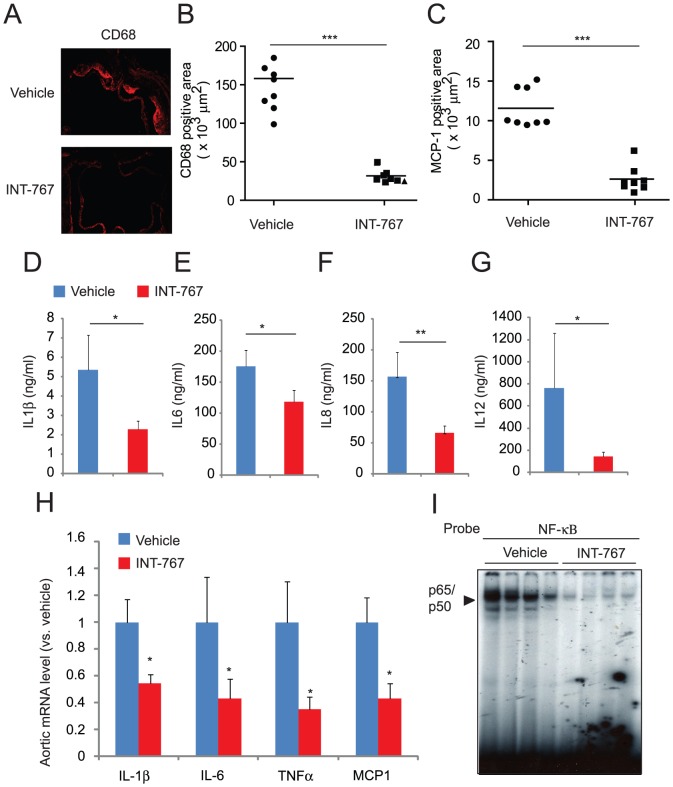
INT-767 inhibits systemic and local inflammation in ApoE^−/−^ mice. Serum A) IL-1β, B) IL-6, C) IL-8 and D) IL-12 levels in ApoE^−/−^ mice treated with INT-767. E) Representative picture of immunofluorescence analysis of CD68-positive macrophages and MCP-1 in the aortas of ApoE^−/−^ mice treated with INT-767. Quantification of immunofluorescence analysis of F) CD68 and G) MCP-1 in the aortas of ApoE^−/−^ mice treated with INT-767. H) mRNA levels of aortic cytokines and chemokines in ApoE^−/−^ mice treated with INT-767. I) NF-κB binding activity in the aortas of ApoE^−/−^ mice treated with INT-767. *P<0.05, **P<0.001 and ***P<0.001.

We next examined whether INT-767 reduced systemic and local inflammation in ApoE^−/−^ mice. ELISA analysis showed that INT-767 treatment reduced levels of circulating cytokines such as IL-1β, IL-6, IL-8 and IL-12 (Fig. 5A–5D). To examine whether dual activation of FXR and TGR5 reduces macrophage infiltration and inflammation in the aorta, we performed immunofluorescence analysis using CD68 as a macrophage marker and monocyte chemotactic protein 1 (MCP-1) as a chemokine. Histological analysis showed a significant reduction of CD68-positive macrophages and MCP-1 in aortic root sections from ApoE^−/−^ mice treated with INT-767 (Fig. 5E, 5F and 5G). In addition, INT-767 significantly reduced aortic expression of inflammatory markers such as IL-1β, IL-6, TNFα and MCP-1 (Fig. 5H). Since NF-κB is a central regulator of inflammation, we examined whether INT-767 affects NF-κB activity in the aorta. EMSA analysis indicated that aortic NF-κB activity was significantly reduced in the aortas of ApoE^−/−^ mice treated with INT-767 (Fig. 5I).

### TGR5 activation but not FXR activation reduces cytokine production in macrophages

**Figure 6:**
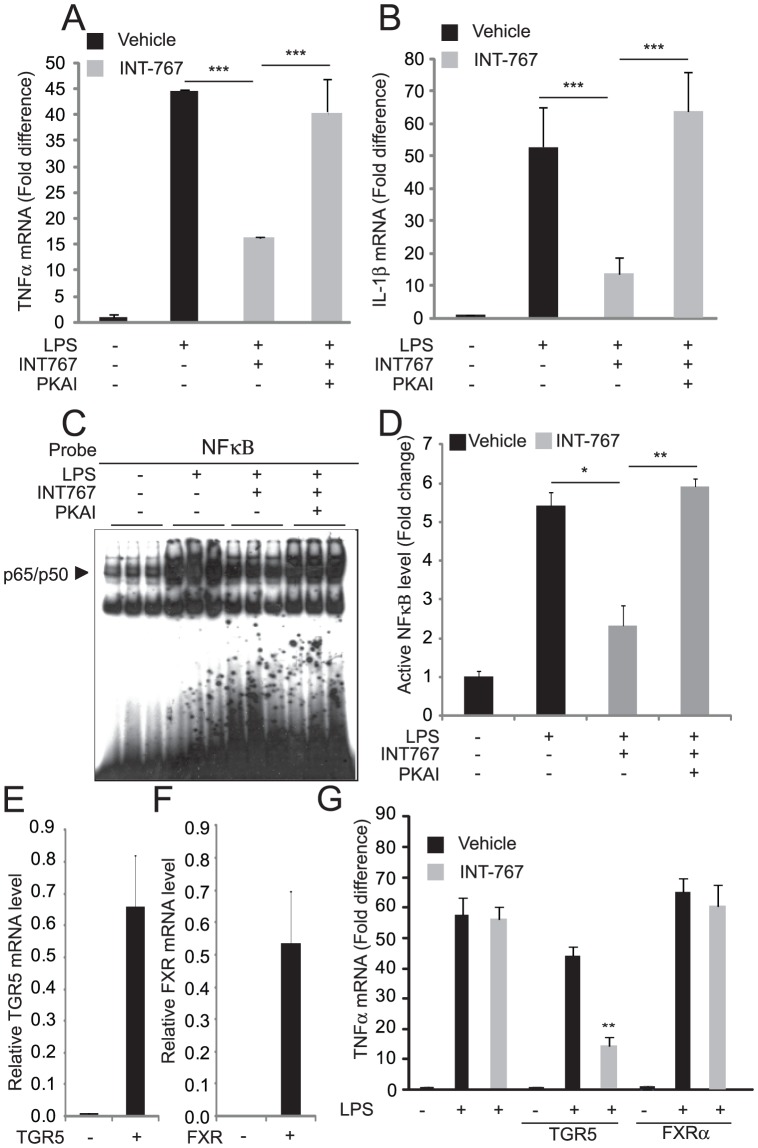
INT-767 inhibits activation of NF-κB and cytokine production through a TGR5-PKA-dependent mechanism. A) TNFα and B) IL-1β mRNA levels, C) NF-κB binding activity by EMSA and D) the densitometric analysis of the NF-κB EMSA. J774.2 macrophages were pretreated with 10 µM INT-767 for 2 hours and treated with LPS (100 ng/ml) for 2 hours in the presence of a PKA inhibitor (1 mM, PKAI, Rp-8-Br-cAMPS). E) TGR5 or F) FXR was overexpressed in Raw294.7 macrophages, which express very low levels of TGR5 and FXR. G) INT-767 inhibits TNFα expression induced by LPS (100 ng/ml) in Raw294.7 macrophages overexpressing TGR5 but not FXR. * P<0.05, **P<0.01, and ***P<0.001.

TGR5 is abundantly expressed in monocytes and macrophages under resting conditions [8]. However, it is not expressed in all macrophage cell lines [8]. To study the effect of TGR5 on cytokine production in macrophages, we first analyzed the levels of TGR5 expression in monocyte and macrophage cell lines including Raw294.7, J774.2, THP-1 and U937 cells. We found that J774.2 cells were the only cells that expressed high levels of TGR5, whereas the other macrophage cell lines expressed very low or undetectable levels of TGR5. We therefore decided to use J774.2 cells to study the effects of INT compounds on LPS-induced cytokine production. INT-767 reduced the induction of cytokine mRNAs such as TNFα and IL-1β in J774.2 cells stimulated with LPS (Fig. 6A and 6B). We next examined whether activation of the cAMP-PKA pathway activated by TGR5 contributes to the inhibition of cytokine expression. PKA inhibitor Rp-8-Br-cAMPS (PKAI) completely blocked the reduction of cytokine production by INT-767. To understand the mechanism by which INT-767 reduced the production of inflammatory cytokines, we performed EMSA analysis to measure the activity of NF-κB, which is a master regulator of cytokine production. EMSA analysis showed that INT-767 treatment significantly reduced levels of active NF-κB p50/p65 complex in the nucleus fraction of NF-κB activity by INT-767 treatment. PKA inhibitor completely blocked the reduction of NF-κB activity by INT-767 (Fig. 6A-6D). INT-767 did not affect LPS-induced cytokine production in Raw294.7 cells, which do not highly express TGR5 or FXR (Fig 6E–G). We next examined whether stable TGR5 (Fig. 6E) or FXR (Fig. 6F) overexpression in Raw294.7 cells could reveal inhibitory effects of INT-767 on cytokine production. When treated with INT-767 and INT-777, Raw294.7 cells that overexpressed TGR5, but not FXR, had lower TNFα expression following LPS treatment (Fig. 6G). These results suggest that the anti-inflammatory effect of INT-767 is due to TGR5 activation, not FXR activation.

## Discussion

Dyslipidemia and chronic inflammation are hallmarks of atherosclerosis. Therefore, identifying a compound that acts to simultaneously limit dyslipidemia and inflammation may prove to be beneficial in reducing atherosclerosis. A number of reports suggest that activation of bile acid signaling pathways can prevent or lessen both dyslipidemia and inflammation through two bile acid receptors, FXR and TGR5 [4], [8], [25]. In this study, we demonstrate that a novel FXR and TGR5 dual agonist, INT-767, potently inhibits atherosclerotic formation by preventing hyperlipidemia and inhibiting pro-inflammatory cytokine production in macrophages.

In addition to reduced serum cholesterol and triglyceride levels, INT-767 reduced serum cholic acid and its metabolites (deoxycholic acid, taurocholic acid and taurodeoxycholic acid), whereas CDCA and tauro-conjugated CDCA levels were unchanged and increased, respectively, in mice treated with INT-767. These data indicate that FXR activation by INT-767 shuts off cholic acid synthesis by reducing the hepatic expression of CYP8B1 (Fig. 3), which determines the balance of CA:CDCA synthesis. In addition, inhibition of cholic acid synthesis by CYP8B1 deficiency inhibits dyslipidemia and atherosclerosis in ApoE^−/−^ mice by decreasing intestinal lipid absorption [26], suggesting that cholic acid deficiency is caused by the lipid-lowering and anti-atherogenic effects of INT-767. Another mechanism by which FXR activation elicits lipid-lowering effects is due to increased liver-dependent cholesterol efflux to feces [20]. We previously reported that FXR activation by synthetic FXR agonists such as Px20606 increased hepatic cholesterol efflux by inducing hepatic genes involved in transhepatic cholesterol efflux such as scavenger-receptor B1 (SR-BI) and ATP-binding cassette subfamily B, member 4 (ABCB4). INT-767 potently induced both hepatic genes (Fig. 3A).

Some bile acids are known to elicit anti-inflammatory effects in macrophages through TGR5 [8], [14]–[15]. In agreement with *in vitro* studies [13], [18], [27]–[28], our *in vivo* study showed that INT-767 treatment reduced circulating inflammatory cytokines such as IL-1β and IL-6 in ApoE^−/−^ mice, and also reduced the expression of inflammatory cytokines and chemokines in the aortas of ApoE^−/−^ mice. INT-767 reduced the LPS-induced expression of inflammatory cytokines from J774.2 macrophages, which highly express TGR5 but not FXR. On the other hand, INT-767 was not able to reduce LPS-induced cytokine production in Raw294.7 macrophages, which express undetectable levels of both TGR5 and FXR. In Raw295.7 cells, TGR5 but not FXR overexpression established the inhibition of cytokine production by INT-767 (Fig 6E–G). These data suggest that the inhibitory effect of INT-767 is due to the activation of TGR5, not FXR. TGR5 activation increases levels of intracellular cAMP, resulting in the activation of PKA. INT-767 increases cAMP levels in bone marrow macrophages [28]. We therefore hypothesized that INT-767 stimulates the production cAMP through TGR5, leading to PKA activation. Consistent with our hypothesis, a PKA inhibitor, RP-8-BR-CAMPS, completely blocked the inhibition of LPS-induced TNFα and IL-1β production by INT-767. This inhibitor is an analog of cAMP and specifically inhibits binding of cAMP to the regulatory I subunit of PKA. These data indicate that INT-767 reduces cytokine production through a cAMP-PKA dependent mechanism. Interestingly, another class of PKA inhibitors, H89, did not affect the inhibitory effect of INT-767 (data not shown). *H89* blocks the activity of *PKA* through competitive inhibition of the ATP binding site on the *PKA catalytic* subunit. A number of reports suggest that PKA type I and type II have distinct effects [29]–[31]. In addition, the phenotypes of mice lacking regulatory subunits of PKA type I and type II are different [32]. These observations suggest that the PKA type I is more important in TGR5-mediated inhibition of cytokine production than PKA type II.

NF-κB is a master regulator of cytokine production in macrophages. INT-767 treatment strongly reduced levels of active NF-κB levels in the aortas of ApoE^−/−^ mice and J774.2 macrophages. Our data indicate that inhibiting both systemic and local inflammation by inactivating the NF-κB pathway contributes to the anti-atherogenic effect of INT-767. In J774.2 cells, RP-8-BR-CAMPS completely blocked the effect of INT-767 on NF-κB activity, suggesting that TGR5 activation inhibits cytokine production through a PKA-dependent mechanism. Although the mechanism by which TGR5-cAMP-PKA inhibits NF-κB activity is still obscure, a recent study showed that several kinase–anchoring proteins (AKAPs) contribute to cAMP-PKA-mediated inhibition of NF-κB and cytokine production in macrophages [30].

In this study, we demonstrated that dual activation of FXR and TGR5 has a beneficial impact on the development of atherosclerosis. The anti-atherogenic effect of INT-767 is due to the combined effects of lowering lipids and inhibiting systemic inflammation. Further studies will be required to examine which receptor contributes to the hypolipidemic, anti-inflammatory and anti-atherogenic effects of INT-767 using knockout mice. Taken together, this study suggests that dual activation of FXR and TGR5 may present a promising strategy for the treatment of atherosclerosis.

## Supporting Information

Figure S1 The NF-κB complex and the activity changes in the aortas of ApoE^-/-^ mice. **A**. DNA-binding results of aortic NF-κB were verified by excess cold probes (left) and antibodies (Ab) that recognize the two components of the classical NF-κB complex, p65 and p50 (right).
(EPS)

## References

[1] Makishima M, Okamoto AY, Repa JJ, Tu H, Learned RM, et al (1999) Identification of a nuclear receptor for bile acids. Science 284: 1362–1365. 10334992 10.1126/science.284.5418.1362

[2] Parks DJ, Blanchard SG, Bledsoe RK, Chandra G, Consler TG, et al (1999) Bile acids: natural ligands for an orphan nuclear receptor. Science 284: 1365–1368. 10334993 10.1126/science.284.5418.1365

[3] Wang H, Chen J, Hollister K, Sowers LC, Forman BM (1999) Endogenous bile acids are ligands for the nuclear receptor FXR/BAR. Mol Cell 3: 543–553. 10360171 10.1016/s1097-2765(00)80348-2

[4] Watanabe M, Houten SM, Wang L, Moschetta A, Mangelsdorf DJ, et al (2004) Bile acids lower triglyceride levels via a pathway involving FXR, SHP, and SREBP-1c. J Clin Invest 113: 1408–1418. 15146238 10.1172/JCI21025 PMC406532

[5] Lee FY, Lee H, Hubbert ML, Edwards PA, Zhang Y (2006) FXR, a multipurpose nuclear receptor. Trends Biochem Sci 31: 572–580. 16908160 10.1016/j.tibs.2006.08.002

[6] Hartman HB, Gardell SJ, Petucci CJ, Wang S, Krueger JA, et al (2009) Activation of farnesoid X receptor prevents atherosclerotic lesion formation in LDLR-/- and apoE-/- mice. J Lipid Res 50: 1090–1100. 19174369 10.1194/jlr.M800619-JLR200 PMC2681391

[7] Mencarelli A, Renga B, Distrutti E, Fiorucci S (2009) Antiatherosclerotic effect of farnesoid X receptor. Am J Physiol Heart Circ Physiol 296: H272–281. 19028791 10.1152/ajpheart.01075.2008

[8] Kawamata Y, Fujii R, Hosoya M, Harada M, Yoshida H, et al (2003) A G protein-coupled receptor responsive to bile acids. J Biol Chem 278: 9435–9440. 12524422 10.1074/jbc.M209706200

[9] Maruyama T, Miyamoto Y, Nakamura T, Tamai Y, Okada H, et al (2002) Identification of membrane-type receptor for bile acids (M-BAR). Biochem Biophys Res Commun 298: 714–719. 12419312 10.1016/s0006-291x(02)02550-0

[10] Thomas C, Gioiello A, Noriega L, Strehle A, Oury J, et al (2009) TGR5-mediated bile acid sensing controls glucose homeostasis. Cell Metab 10: 167–177. 19723493 10.1016/j.cmet.2009.08.001 PMC2739652

[11] Watanabe M, Houten SM, Mataki C, Christoffolete MA, Kim BW, et al (2006) Bile acids induce energy expenditure by promoting intracellular thyroid hormone activation. Nature 439: 484–489. 16400329 10.1038/nature04330

[12] Li T, Holmstrom SR, Kir S, Umetani M, Schmidt DR, et al (2011) The G protein-coupled bile acid receptor, TGR5, stimulates gallbladder filling. Mol Endocrinol 25: 1066–1071. 21454404 10.1210/me.2010-0460 PMC3100601

[13] Pols TW, Nomura M, Harach T, Lo Sasso G, Oosterveer MH, et al (2011) TGR5 activation inhibits atherosclerosis by reducing macrophage inflammation and lipid loading. Cell Metab 14: 747–757. 22152303 10.1016/j.cmet.2011.11.006 PMC3627293

[14] Ichikawa R, Takayama T, Yoneno K, Kamada N, Kitazume MT, et al. (2012) Bile acids induce monocyte differentiation toward IL-12 hypo-producing dendritic cells via a TGR5-dependent pathway. Immunology. 10.1111/j.1365-2567.2012.03554.x PMC3403261 22236403

[15] Keitel V, Donner M, Winandy S, Kubitz R, Haussinger D (2008) Expression and function of the bile acid receptor TGR5 in Kupffer cells. Biochem Biophys Res Commun 372: 78–84. 18468513 10.1016/j.bbrc.2008.04.171

[16] Ceryak S, Bouscarel B, Malavolti M, Robins SJ, Caslow KL, et al (2000) Effect of ursodeoxycholic acid on hepatic LDL binding and uptake in dietary hypercholesterolemic hamsters. Atherosclerosis 153: 59–67. 11058700 10.1016/s0021-9150(00)00396-8

[17] Sehayek E, Ono JG, Duncan EM, Batta AK, Salen G, et al (2001) Hyodeoxycholic acid efficiently suppresses atherosclerosis formation and plasma cholesterol levels in mice. J Lipid Res 42: 1250–1256. 11483626

[18] Rizzo G, Passeri D, De Franco F, Ciaccioli G, Donadio L, et al (2010) Functional characterization of the semisynthetic bile acid derivative INT-767, a dual farnesoid X receptor and TGR5 agonist. Mol Pharmacol 78: 617–630. 20631053 10.1124/mol.110.064501 PMC2981390

[19] Miyazaki-Anzai S, Levi M, Kratzer A, Ting TC, Lewis LB, et al. (2010) FXR activation prevents the development of vascular calcification in ApoE^−/−^ mice with chronic kidney disease. Circ Res: 1807–1817. 10.1161/CIRCRESAHA.109.212969 PMC2909765 20431060

[20] Hambruch E, Miyazaki-Anzai S, Hahn U, Matysik S, Boettcher A, et al (2012) Synthetic farnesoid X receptor agonists induce high-density lipoprotein-mediated transhepatic cholesterol efflux in mice and monkeys and prevent atherosclerosis in cholesteryl ester transfer protein transgenic low-density lipoprotein receptor (-/-) mice. J Pharmacol Exp Ther 343: 556–567. 22918042 10.1124/jpet.112.196519 PMC11047796

[21] Lim RS, Kratzer A, Barry NP, Miyazaki-Anzai S, Miyazaki M, et al (2010) Multimodal CARS microscopy determination of the impact of diet on macrophage infiltration and lipid accumulation on plaque formation in ApoE-deficient mice. J Lipid Res 51: 1729–1737. 20208058 10.1194/jlr.M003616 PMC2882730

[22] Miyazaki M, Kim YC, Gray-Keller MP, Attie AD, Ntambi JM (2000) The biosynthesis of hepatic cholesterol esters and triglycerides is impaired in mice with a disruption of the gene for stearoyl-CoA desaturase 1. J Biol Chem 275: 30132–30138. 10899171 10.1074/jbc.M005488200

[23] Ando M, Kaneko T, Watanabe R, Kikuchi S, Goto T, et al (2006) High sensitive analysis of rat serum bile acids by liquid chromatography/electrospray ionization tandem mass spectrometry. J Pharm Biomed Anal 40: 1179–1186. 16242877 10.1016/j.jpba.2005.09.013

[24] Liu X, Miyazaki M, Flowers MT, Sampath H, Zhao M, et al (2010) Loss of Stearoyl-CoA desaturase-1 attenuates adipocyte inflammation: effects of adipocyte-derived oleate. Arterioscler Thromb Vasc Biol 30: 31–38. 19910642 10.1161/ATVBAHA.109.195636 PMC2837593

[25] Zhang Y, Yin L, Anderson J, Ma H, Gonzalez FJ, et al (2010) Identification of novel pathways that control farnesoid X receptor-mediated hypocholesterolemia. J Biol Chem 285: 3035–3043. 19996107 10.1074/jbc.M109.083899 PMC2823426

[26] Slatis K, Gafvels M, Kannisto K, Ovchinnikova O, Paulsson-Berne G, et al (2010) Abolished synthesis of cholic acid reduces atherosclerotic development in apolipoprotein E knockout mice. J Lipid Res 51: 3289–3298. 20675645 10.1194/jlr.M009308 PMC2952569

[27] Pellicciari R, Gioiello A, Macchiarulo A, Thomas C, Rosatelli E, et al (2009) Discovery of 6alpha-ethyl-23(S)-methylcholic acid (S-EMCA, INT-777) as a potent and selective agonist for the TGR5 receptor, a novel target for diabesity. J Med Chem 52: 7958–7961. 20014870 10.1021/jm901390p

[28] McMahan RH, Wang XX, Cheng LL, Krisko T, Smith M, et al (2013) Bile acid receptor activation modulates hepatic monocyte activity and improves nonalcoholic fatty liver disease. J Biol Chem 288: 11761–11770. 23460643 10.1074/jbc.M112.446575 PMC3636865

[29] Raskovalova T, Lokshin A, Huang X, Su Y, Mandic M, et al (2007) Inhibition of cytokine production and cytotoxic activity of human antimelanoma specific CD8+ and CD4+ T lymphocytes by adenosine-protein kinase A type I signaling. Cancer Res 67: 5949–5956. 17575165 10.1158/0008-5472.CAN-06-4249

[30] Wall EA, Zavzavadjian JR, Chang MS, Randhawa B, Zhu X, et al (2009) Suppression of LPS-induced TNF-alpha production in macrophages by cAMP is mediated by PKA-AKAP95-p105. Sci Signal 2: ra28. 19531803 10.1126/scisignal.2000202 PMC2770900

[31] Torgersen KM, Vang T, Abrahamsen H, Yaqub S, Tasken K (2002) Molecular mechanisms for protein kinase A-mediated modulation of immune function. Cell Signal 14: 1–9. 11747983 10.1016/s0898-6568(01)00214-5

[32] Chin KV, Yang WL, Ravatn R, Kita T, Reitman E, et al (2002) Reinventing the wheel of cyclic AMP: novel mechanisms of cAMP signaling. Ann N Y Acad Sci 968: 49–64. 12119267 10.1111/j.1749-6632.2002.tb04326.x

